# The evaluation of the benefits of pain control by patients using PCA pump compared to medicine injection to ease the pain by nurses

**Published:** 2015

**Authors:** MH Nemati

**Affiliations:** * Hospital, Shiraz, Iran Ordibehesht

**Keywords:** pain control, PCAT Pump, NCA

## Abstract

Pain is considered as the fifth vital sign, and the need to control pain after surgery emphasizes its importance. Pain after surgery leads to unpleasant outcomes and effects on different mechanisms thus causes fundamental changes in the metabolism of the body of susceptible people. Accordingly, the current article aimed to compare two methods of pain control by the patient using PCA pumps compared to medicine injection to ease the pain by nurses.

**Methodology:** In the current research, a single-blind clinical trial of 70 patients as nominees of undergoing open-heart surgery at Shiraz Ordibehesht Hospital during 12 months was examined. The patients randomly divided into two groups of 35 patients with pain control by analgesia pumps of PCA and by NCA. The pain intensity was analyzed by visual analog scale (VAZ) and the sedation degree was examined based on the factors such as the Richmond sedation, nausea and vomiting and respiratory depression induced by opioids and analgesic effects, arterial oxygen saturation, patient satisfaction, ventilation duration for up to 48 hours after surgery. For data analysis, statistical software SPSS was used.

**Results:** No statistically clear distinction was seen in the status of nausea and vomiting as well as in the length of ventilation and the oxygen saturation status between the two groups. In none of the two groups, respiratory depression was observed. There was a statistically notable variation between the two teams regarding satisfaction level. Also, an increasing significant decline in pain intensity was noted in both groups at consecutive times. There was an experimentally meaningful distinction between the two teams regarding degree of sedation (P < 0.001), such that the level of sedation in NCA group was higher (P < 0.001).

**Conclusion:** The use of PCA pump in acute pain control after open cardiac surgery was better than the NCA. In the case of using the PCA pump, in addition to the psychological effect, the pump could control pain. Thus, the side effects of high dose injections would be reduced, and the increased use of the dose of drugs would not be detected.

## Introduction

Pain is considered as the fifth vital sign, and the need to control it after surgery emphasizes its importance. Pain after surgery leads to unpleasant outcomes and affects different mechanisms, thus causing fundamental changes in the metabolism of the body of the susceptible people.

Given the large open-heart surgery and the extreme anatomical and physiological changes that are unique in their kind, patients after surgery undergo severe and intolerable pains due to cuts in skin, muscle and bone that are especially done on the chest. Pain can not only lead to the recurrence of a heart attack but may also cause problems such as the reduction in the operational capacity of the lungs, the lack of an effective cough due to the pain and subsequently lung secretions accumulation, the atelectasis, pneumonia, and lung infection. Also, it may lead to tachycardia, hypertension, cardiac arrhythmias, thrombotic complications like (DVT), pulmonary embolism, pain, anxiety, insomnia, fear, dissatisfaction of patients with the operation, nausea, repeated vomiting, constipation, and flatulence, which should be eased by analgesics and particularly opioids. In pain management by nurses, high dose of narcotic medications is injected in the form of a bolus that causes some complications such as respiratory depression, hypotension, nausea, and vomiting.

An effective method of pump pain relief is the use of PCA (Patient Controlled Analgesia), which includes parts of a bolus dose, the required dose, and the intervals when the pump is locked, and continuous injection of a tranquilizer [**1**]. PCA pumps operates the same as a lock and prevent excessive doses of drugs from entering the body. The patient pushes the control button to get the drug into the body, but the pump only injects the programmed amount [**2**]. In using the PCA pump, due to the continued injection of a little amount of drug (yet in a continuous manner), the beneficial effects of opioid analgesics can be observed, while their side effects are not observed. 

Sima Lakdizaji et al. (2012) carried out a research entitled “the impact of pain control method by the patient on the relief of postoperative pain of coronary artery bypass surgery”. The results indicated that PCA method, with continuous infusion of morphine, leads to an increase in morphine consumption and a reduction in patient’s pain, and it seems that this method would be more efficient than NCA, and PCA method would be used in coronary artery graft bypass surgery [**3**]. Daniel et al. (2005) presented a precise study and meta-investigation to evaluate whether or not PCA has a better clinical outcome than NCA. The results showed that in patients undergoing cardiac surgery by PCA method, the cumulative dose of morphine could be increased in 24-48 hours and therefore result in an improvement in VAS 48 hour compared to the NCA [**4**]. Also, the research conducted by Peterson et al. (2000) revealed that the treatment with PCA after coronary vein detour grafting surgery is preferably better in the treatment of the pain and more use of drugs externally an improvement in side impacts, related to conventional methods of pain control by NCA [**5**].

The current research aimed to analyze pain control in two groups of PCA and NCA in the patients of Ordibehesht Hospital in Shiraz. 

## Materials and methods

The present research was an interventional study and of a type of single-blind interventional randomized clinical trial, that tried to examine the benefits of pain control by using the PCA pump compared to drug injection to ease the pain by nurses.

The target population included all the patients undergoing open-heart surgery during the 12-months period in 2015, who underwent the surgery in Ordibehesht Hospital in Shiraz. Exclusion criteria included: age more than seventy years, BUN ˃ 40 mg/ dl, Cr ˃ 2mg/ dl, EF < 30% (poor left ventricular function), drug allergy, drug or alcohol addiction, and withdrawal patients every time. In this study, 70 patients were randomly divided into two groups of 35 people of PCA and 35 people of NCA.

In the control group, after being extubated, the patient’s expression of pain is controlled by routine medication injection procedures and by a nurse, and in PCA, the pain is controlled by continuous injections and bolus of PCA device.

In PCA group, one day before the surgery, the patient was informed of the use of pumps and his consent to perform such act asked, and he was also trained how to push the button related to the pain pump. In this group, when the patient felt a new pain, he could press the button, and the pump infused an additional pre-determined dose of the bolus to the patient, and the patient’s pain reduced in this way.

In this group, immediately after surgery and by entering the ICU, the pain pump was placed for 48 hours. The volume of PCA pump (Jiaxing u-life medical device technology co., Ltd) was 100cc and lock time set 15 minutes, the duration of which was designed to prevent the patient’s overdose. Also, the infusion rate 4cc/ hr and bolus dose of 0.5 ccs/ every time (bolus dose by each time pushing the button is 0.5cc) were formulated. The pump contained morphine, midazolam, Apostle, and Ketamin. 

The dose of medication was such that in the first 24 hours, morphine 10 mg, midazolam 5 mg, Apostle 3 grams, 50 mg ketamine; and in the second 24 hours, morphine 5 mg, midazolam 5 mg, Apostle 2 grams, and ketamine 25 mg. Each one cc of the pump solution, at the first 24-hour, contained 0.1 mg of morphine, 0/ 05 mg of midazolam, 30 mg Apostle, and 0.5 Ketamine. In the 24 hours, each single cc of the pump solution included 0.05 mg morphine, 0.05 mg midazolam, 20 mg Apostle, and 0.25 mg ketamine.

In NCA group, the patient’s pain was controlled by the nurse. Whenever the patient felt pain, the nurse administered analgesic medication as an IV based on the ward’s routine. The routine of the ICU ward of Ordibehesht Hospital used midazolam, diazepam, morphine, pethidine, Apostle, and the use of NSAID's medications. The nurses administered one of these drugs for pain relief based on the pain intensity. When the patient was on a ventilator, some medications such as midazolam, diazepam, morphine, pethidine were administered, and when the patient was intubated, Apostle and NSAIDs were used.

In both groups, the used anesthetic medications were the same and included Midazolam, Morphine, Pavilion, Fentanyl, and Pentothal. 

The table on the visual analog pain scale (VAS) was based on some shape presented to the patients and patients expressed the degree of their pain and then they were recorded. The patients were asked about the amount of the pain every 6 hours of extubation until they admitted to ICU. Some recommendations were presented to the patients regarding the research at hand and how the visual scale analyzed the pain, and they were asked to reply to some questions on pain at regular intervals after surgery. 

VAS ranges from 0 to 10; in which 0 is no pain, and 10 is the maximum pain, i.e. the worst pain felt by the patient. The degree of patient’s sedation in PCA group was measured every six hours. 

The variables of the present research were nausea and vomiting, duration of ventilation, visual analog pain scale (VAS), sedation rate, artery oxygen saturation, and respiratory depression, and also patient satisfaction about the amount of pain that. In this study, the patient’s satisfaction was gained by orally asking some questions. Based on the questions, the patients were to rate their satisfaction on the tolerable level of pain, and the replies ranged from bad, not good, average, good and very good.

Sample size: according to the objectives and the type of the study, and based on the previous research in this regard, and also considering assumptions that included an error of 5%, capability of 80%, and the effect rate of 60%, using the formula,

$$n=\;\frac{2s^{2}{\left(t_{\alpha ,v}+t_{\beta \left(1\right),v}\right)}^{2}\;}{{\left(\partial \right)}^{2}}$$

32 patients in each group were assigned. Given the longitudinal type of the research and repeated measures by using the formula, $n^{\prime }=n\;\times \;\frac{1}{1-p}$ and the losing of 10%, and the final sample size that was 35 in each group, a total of 70 patients being needed.

Block design permutations were used as a randomized method. After selecting each sample, the sample was assigned to treatments A and B by using the random number table. The numbers 0 to 4 were assigned to AB permutation, and 5 to 9 assigned to BA permutation. After completing a questionnaire and its thorough investigation, the data were inserted in the form of code in Code Sheet. The existing problems were rectified by using a review of the survey. Then, the coded data were entered into SPSS. After preparing data in SPSS, the data were once considered to ensure their authenticity.

Data analysis was done by using descriptive statistics methods and independent t-test and analysis of variance with repeated measures, chi-square tests, McNemar’s tests, or nonparametric tests at 5% level of significance. To analyze the data, EXCELL and SPSS statistical software was used.

## Results

N this study, 70 patients in two groups of 35-subject PCA and NCA were reviewed. For comparing the Nausea/ Vomit (N/ V) in both groups, Chi-square was used. Based on this analysis, 45.7% of PCA and 54.3% of the NCA, had positive N/ V. However, the observed difference was not considered statistically significant (P = 0.473) **Table 1**.

**Table 1:** Situation of NV at 2 teams under study

	Variable	Group		P-value
		PCA (n= 35)	NCA (n= 35)	
N/V	Positive	16 (45.7)	19 (54.3)	0.473
	Negative	19 (54.3)	16 (45.7)	
	Total	35 (100)	35 (100)	

**Fig. 1:**
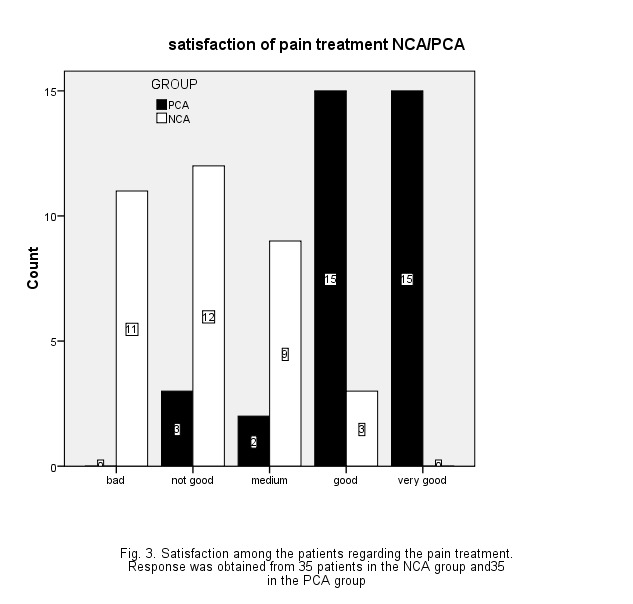
The frequency distribution of patients according to their satisfaction

There was no statistically significant difference regarding the status of nausea and vomiting of the two groups. To compare the satisfaction score in the two groups, Mann-Whitney U Test was used. Based on this test, a statistically significant difference was observed between the satisfaction scores of the two groups of patients (P < 0.001) (**Table 2**, **Fig. 1**).

**Table 2:** A comparison of the patients’ satisfaction scores in the two groups under study

P-value	Group		Variable
	NCA ± sd Mean	PCA ± sd Mean	
<0.001	0.96 ± 0. 11	0.9 ± 2. 20	Satisfaction Score

To compare the length of ventilation (L/ V)) in two groups, a t-test was used. Accordingly, there was no clear distinction between the 2 teams (P = 0.401) (**Table 3**).

**Table 3:** A comparison of the length of ventilation in the two study groups

Variable	Group		P-value
	PCA ± Mean(SD)	NCA ± Mean(SD)	
Length of Ventilation	1.9 ± 7. 6	2.09 ± 7. 2	0.401

**Fig. 2:**
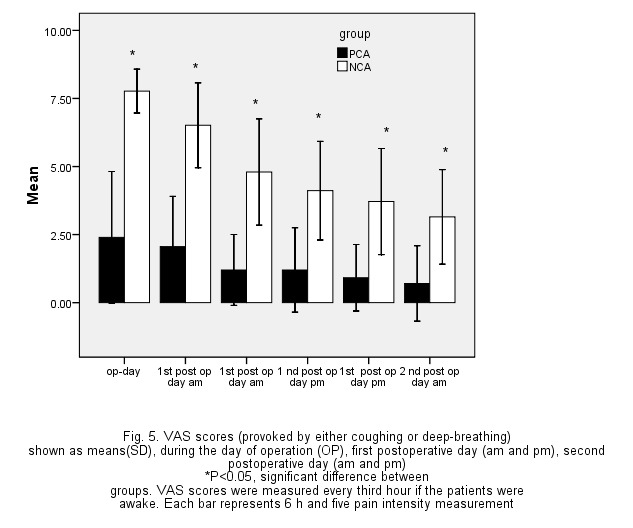
The mean (standard deviation) of the scores of VAS in the study groups

**Fig. 3:**
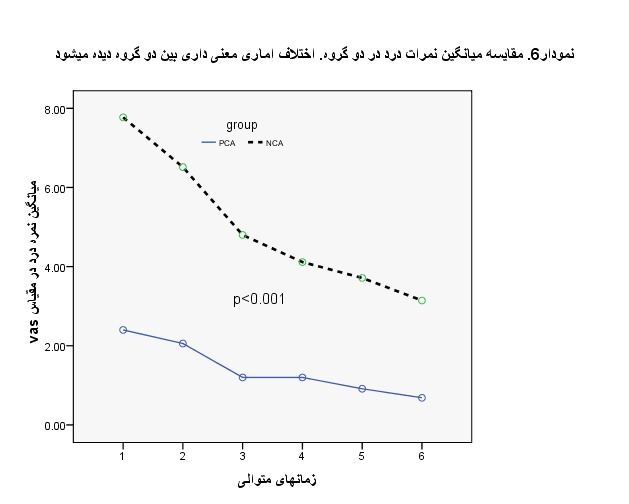
A comparison of the mean pain scores of the study groups

To compare the score VAS (Visual analog scale pain), at various times between the two groups, the analysis of variance with repeated measures was used, the analysis results being given in **Table 4**. Based on the results, a statistically clear distinction was observed between the 2 teams. Also, the difference between the two groups at different times was obtained based on the t-test, and at all times there was a statistically clear variation between the 2 teams (P < 0.001), and PCA group had lower scores than the NCA (**Fig. 2**). Also, a significant decline in pain intensity was observed in both groups at consecutive times (**Table 4**, **Fig. 3**).

**Table 4:** The mean pain score on the scale and in the two groups

Consecutive Times		Group		P-value
	PCA ± Mean(SD)	NCA ± Mean(SD)		
1st day	18-24	2.4 ± 2.4	0.8± 7. 77	<0.001
2nd day	24-6	1.8 ± 2.05	1.56± 6. 51	<0.001
	6-12	±1.3 1.2	1.95± 4. 8	<0.001
	12-18	1.5±1.2	1.8± 4. 11	<0.001
	18-24	1.22± 0. 91	1.94± 3. 7	<0.001
3rd day	24-6	1.3± 0. 68	1.7± 3.14	
P-value	<0.001	<0.001		

**Fig. 4:**
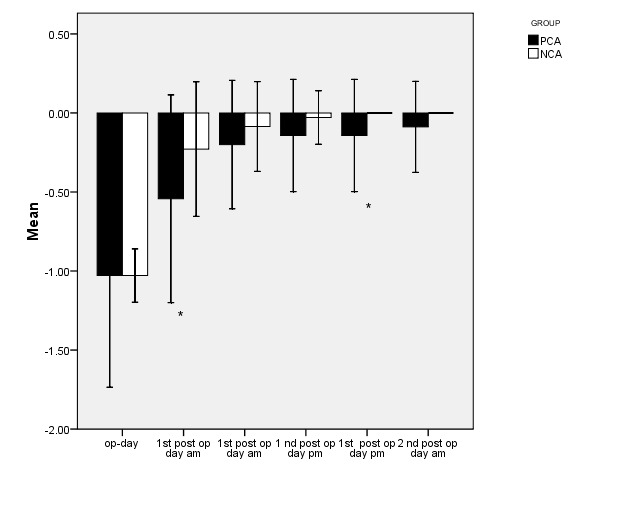
Mean amount of sedation in the two study groups

To compare the degree of sedate in the two groups, the analysis of variance with repeated measures was used. The findings indicated that there was a clear difference between the 2 teams (P < .001). Also, at various times, the difference between the two groups was compared by using a t-test, whose difference was not initially significant, but it was great at time 2 (1st post op day am) and 5 (1st post op day pm) was significant) (**Table 5**, **Fig. 4**).

**Table 5:** Average amount of sedation in the two groups

Consecutive Times		Group		P-value
	PCA ± Mean(SD)	NCA ± Mean(SD)		
1st day	18-24	-0.7±1.02	-0.16±1.02-	1
2nd day	24-6	-0.56±0.54	-0.42±0. 22	0.02
	6-12	-0.40±0.20	-0.28± 0. 08	0.177
	12-18	-0.25±0.14	-0.16± 0. 02	0.09
	18-24	-0.35±0.14	0	0.02
3rd day	24-6	-0.24±0.38	0	
P-value	<0.001	<0.001		

According to the analysis of variance and the results of the test, the trend of both groups significantly increased (P < 0.001). However, significant differences could be observed between the two groups, so that the degree of the sedate in the group NCA was at a higher state (P < 0.001).

A diagram was used to show the trend of changes in the degree of sedation in both groups. Based on this diagram, in both groups, NCA and PCA had an increasing trend, while changes in PCA were at a lower level. 

**Fig. 5:**
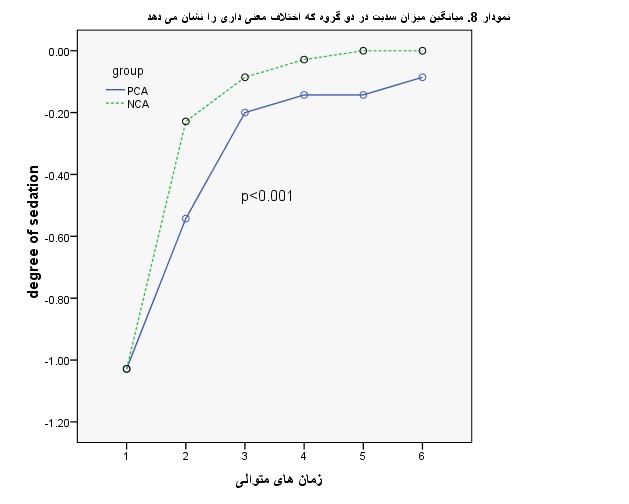
Mean amount of sedate at both groups

**Fig. 6:**
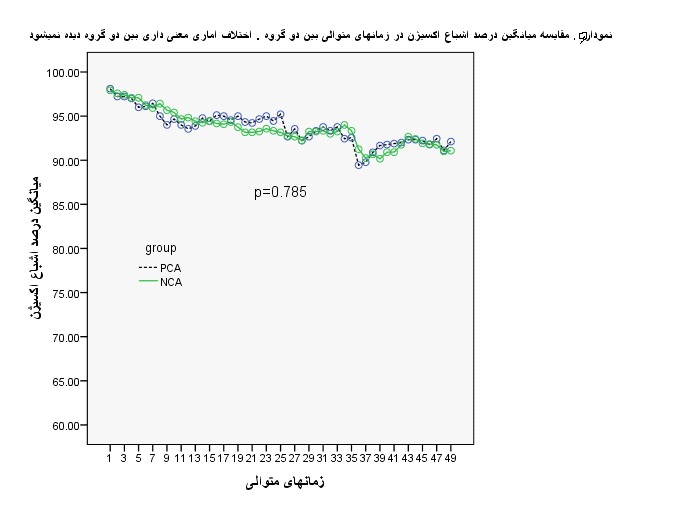
A comparison of the mean amount of the percentage of oxygen saturation at the consecutive times between the two groups

To compare the percentage of oxygen saturation in the group, ANOVA test was used with repeated measures. The results revealed that there was not a statistically clear variation between the 2 teams. This meant that at any time, no clear distinction was observed between the 2 teams, and both groups were statistically identical all the time (**Fig. 5**,**6**).

In none of the two groups, respiratory depression was observed. The length of stay in ICU was of 48 hours, and the duration of hospital stay was of five days; this was identical and did not differ in the two groups.

## Conclusion

Finally, it was found that the application of the PCA pump and keeping on infusion via small amounts of drugs after open-heart surgery could improve pain control and the side effects of drugs and an increased dose of drugs would not be seen.
